# Enhanced Reactive Blue 4 Biodegradation Performance of Newly Isolated *white rot fungus Antrodia P5* by the Synergistic Effect of Herbal Extraction Residue

**DOI:** 10.3389/fmicb.2021.644679

**Published:** 2021-03-30

**Authors:** Tianjie Yuan, Shuyi Zhang, Yifei Chen, Ran Zhang, Letian Chen, Xiaoshu Ruan, Sen Zhang, Fang Zhang

**Affiliations:** ^1^School of Pharmacy, Nanjing University of Chinese Medicine, Nanjing, China; ^2^Jiangsu Collaborative Innovation Center of Chinese Medical Resources Industrialization, Nanjing University of Chinese Medicine, Nanjing, China

**Keywords:** biodegradation, white rot fungus, herbal extraction residues, anthraquinone dyes, reactive blue 4

## Abstract

In this study, a white rot fungus *Antrodia* was newly isolated and named P5. Then its dye biodegradation ability was investigated. Our results showed that P5 could effectively degrade 1,000 mg/L Reactive Blue 4 (RB4) in 24 h with 95% decolorization under shaking conditions. It could tolerate a high dye concentration of 2,500 mg/L as well as 10% salt concentration and a wide range of pH values (4–9). Herbal extraction residues (HER) were screened as additional medium elements for P5 biodegradation. Following the addition of *Fructus Gardeniae* (FG) extraction residue, the biodegradation performance of P5 was significantly enhanced, achieving 92% decolorization in 12 h. Transcriptome analysis showed that the expression of multiple peroxidase genes was simultaneously increased: Lignin Peroxidase, Manganese Peroxidase, Laccase, and Dye Decolorization Peroxidase. The maximum increase in Lignin Peroxidase reached 10.22-fold in the presence of FG. The results of UV scanning and LC-HRMS showed that with the synergistic effect of FG, P5 could remarkably accelerate the biodegradation process of RB4 intermediates. Moreover, the fungal treatment with FG also promoted the abatement of RB4 toxicity. In sum, white rot fungus and herbal extraction residue were combined and used in the treatment of anthraquinone dye. This could be applied in practical contexts to realize an efficient and eco-friendly strategy for industrial dye wastewater treatment.

## Introduction

Synthetic reactive dyes are one of the most consumed chemicals in various industries such as textiles, leathers, pharmaceuticals, and cosmetics (Varjani et al., 2020). It has been estimated that over 700,000 tons of dyes are produced globally every year (Katheresan et al., 2018). However, most of the synthetic dyes are untreated and discharged into water bodies. The amount of discharge of untreated dyes is substantial and seriously threatens ecosystems and human health.

Anthraquinone dyes are the second largest group after azo dyes and are widely used in commercial and industrial applications (Sugano et al., 2009; Becelic-Tomin et al., 2014). Anthraquinone dyes are derived from anthraquinone and have a quinoid ring as the chromophore. The fused nature of polymeric benzene rings makes these dyes more toxic and recalcitrant compared to other commercial dyes (Zeng et al., 2011; Chaudhari et al., 2017; Li et al., 2019). Reactive Blue 4 (RB4) is one of the representative anthraquinone dyes widely used in the textile industry, which is not only hazardous to the ecological environment, but also mutagenic to human cells (Binupriya et al., 2010). There is an urgent need for eco-friendly treatment of industrial anthraquinone dyes prior to their discharge into the environment.

Various physical and chemical methods have been developed to remove and mineralize dyes from industrial effluent in recent years (Holkar et al., 2016). However, conventional methods such as absorption, photo-oxidation, and coagulation-flocculation are generally costly and ineffective (Schrank et al., 2004; Gadekar and Ahammed, 2016; Tang et al., 2018). In comparison, biological methods have attracted significant attention because they are cost-effective and non-hazardous (Jamee and Siddique, 2019). White rot fungus is frequently applied in the bioremediation of anthraquinone dyes due to its relatively robust dye tolerance, as well as its high ability to secrete several extracellular oxidoreductase enzymes including Lignin peroxidase (LiP), Manganese peroxidase (MnP), Dye decolorization peroxidase (DyP), and Laccase (Lac), which facilitates the effective biodegradation of anthraquinone dyes (Gao et al., 2010; Dauda and Erkurt, 2020). In addition to their use in cell-based biological treatments, enzymes are also excellent biocatalysts in dye biodegradation. Recently, the oxidoreductase enzyme Laccase has been immobilized by different methods such as the cross-linked protein–metal hybrid nanoflower system (Patel et al., 2019) and Fe_2_O_3_ yolk-shell particles (Patel et al., 2016), which facilitate repeated enzyme use and effective application in dye and toxic organic biodegradation.

Due to the clear benefit in disease prevention and treatment, the demand for traditional herbal medicine has significantly increased in recent years (Xi et al., 2015). Additionally, herbal extracts are also widely used in synthesis and biotechnological applications. Biomolecules from the extract of green tea leaves were encapsulated with SiO_2_ for the synthesis of silver nanoparticles as effective catalysts (Otari et al., 2019). The leaf extract of *Canna edulis* has also been used to synthesize silver nanoparticles, showing excellent antimicrobial activity against various pathogens (Otari et al., 2017). It is estimated that over 1.5 million tons of herbal extraction residues (HER) are discharged annually in China following conventional extraction (Liu and Cheng, 2009). This puts huge pressure on the ecological environment and hence there is a pressing need to recycle these waste resources. However, there is a dearth of research into effective HER utilization. Hitherto, the reutilization of these resources has tended to focus on the re-extraction of other ingredients or conversion to animal feedstuff (Li et al., 2014; Wang et al., 2017). Several studies have reported that HER can be used as culture media for microbe fermentation and stimulate the production of extracellular enzymes. Extraction residues of *Glycyrrhiza uralensis Fisch* were applied in the production of succinic acid by genetically engineered *E. coli* (Wang et al., 2018). *Penicillium oxalicum* G2 can grow on *Radix isatidis* residues, and exhibits high FPase, cellulose, and beta-glucosidase activities (Zhang S. et al., 2019). *Aspergillus oryzae* can ferment herbal extraction residues to increase antioxidant and antimicrobial activities (Wen et al., 2013). However, the potential efficacy of HER in dye wastewater treatment by white rot fungus has not previously been explored.

In the present study, white rot fungus P5 was isolated and its ability to biodegrade RB4 was investigated. Further, the enhancement of RB4 biodegradation by concomitant utilization of HER was explored. A possible mechanism explaining this enhancement is proposed based on the results of comparative transcriptome analysis. The biodegradation products and toxicological effect of RB4 were also analyzed. Overall, white rot fungus and HER were combined and used in the bioremediation of anthraquinone dyes, which not only benefits in terms of the comprehensive utilization of herbal extraction residue but also provides a new strategy for dye wastewater treatment.

## Materials and Methods

### Reagents and Materials

The following synthetic dyes were purchased from Sangon Biotech Co., Ltd. (Shanghai, China): Reactive Blue4, Methyl Orange, Malachite Green, Reactive Black 19, and Acid Red. They all had a purity exceeding 95%. Other fine chemicals (analytical grade) were purchased from Macklin Biochemical Technology Co., Ltd. (Shanghai, China). Biochemical reagents were purchased from TaKaRa Biotechnology Co., Ltd. (Dalian, China).

### Preparation of Herbal Extract Residues

Four different Chinese herbal medical materials were purchased from the local drugstore: *Fructus Gardeniae* (FG), *Glycyrrhiza uralensis* (GU), *Forsythia suspensa* (FS), and *Sophora alopecuroides* (SA). They were cut into pieces and ultrasound extracted with methanol (1:5 v/v) three times to remove bioactive ingredients and pigments. The herbal extraction residues were first dried then milled in a vegetation disintegrator and separated by passing through a 40-mesh screen. Before use, the HER raw materials were dried at 80°C to a constant weight (Zhao and Zhou, 2016).

### Strain Isolation, Identification, and Culture Conditions

White rot fungus P5 was isolated from the soil of the Chinese herbal garden on the campus of Nanjing University of Chinese Medicine. Potato Dextrose Agar (PDA) was used to screen for the white rot fungus and Potato Dextrose Broth (PDB) was used as liquid media for fungal culture, which was sterilized at 115°C for 30 min before inoculation. The fungal culture conditions were 30°C, 160 rpm/min.

A fungal genome extraction kit (Sangon) was used to extract the fungal genome of P5. Molecular identification involved amplifying 18S rDNA primers which were then sent for sequencing at Genscript Biotech Co., Ltd. (Nanjing, China).

### Biodegradation Assessment of Synthetic Dye

The dye decolorization experiments were performed in 250 ml flasks with 100 ml PDB. The fresh mycelium of P5 on PDA was washed into PDB using sterile water, which was cultured for 2 days as the seed liquid. The inoculum was 5% (v/v) and culture flasks were kept in shaking conditions for 2 days. Different dyes were added to these cultures with initial concentrations of 1,000 mg/L.

Five different synthetic dyes were used to investigate the biodegradation capacity of P5. The maximum absorbance of these dyes are as follows: RB4 (λ_max_ = 595 nm), Methyl Orange (λ_max_ = 460 nm), Malachite Green (λ_max_ = 620 nm), Reactive Black 19 (λ_max_ = 600 nm), and Acid Red (λ_max_ = 520 nm). Sample aliquots were collected at an equivalent time interval of every 24 h until 96 h. Collected samples were centrifuged at 12,000 rpm for 10 min. The supernatant was collected and the maximum absorbance of different dyes was measured by a Shimadzu UV-2400 spectrophotometer. The percentage of dye removed was calculated using the following equation: Decolorization percentage (%) = (A_0_–A)/A_0_^∗^100%, where A_0_ represents the initial absorbance and A is the absorbance after decolorization (Neetha et al., 2019).

### The Effect of Different Parameters on RB4 Degradation

The following parameters were investigated: pH, initial dye concentrations, salt concentration, carbon sources, and nitrogen sources (Bankole et al., 2018). PDB (pH = 6, natural pH value) was adjusted by adding HCl and NaOH to investigate the effect of different pH values (4, 5, 7, 8, and 9) on biodegradation performance. PDB was supplemented with NaCl (0, 10, 20, 30, and 40 g/L) to investigate the salt tolerance of P5. Different initial RB4 dye concentrations (1000, 1500, 2000, and 2500 mg/L) were investigated to explore the dye tolerance of P5. Different carbon sources including maltose, sucrose, fructose, and lactose, as well as nitrogen sources including soybean meal, peptone, yeast extract, and ammonium sulfate (10 g/L, 1%), were used to explore the effects of nutrition on dye biodegradation. The degradation samples were collected every 24 h and centrifuged at 12,000 rpm for 10 min. The supernatant of absorbance was measured for dye decolorization. All these experiments were performed in triplicate.

### The Effect of Different HER on RB4 Degradation

Four different HERs including *Fructus Gardeniae* (FG), *Glycyrrhiza uralensis* (GU), *Forsythia* (F), *Sophora alopecuroides* (SA) were added into the PDB (1%, v/m), which were sterilized at 115°C for 30 min before inoculation. The flasks were inoculated with P5 (5%, v/v) and cultured at 30°C under shaking conditions of 160 rpm/min. Samples were collected every 12 h and centrifuged at 12,000 rpm for 10 min. The supernatant of absorbance was measured for decolorization. All these experiments were performed in triplicate.

### Transcriptome Response of P5 Under FG Stimulation

The prepared P5 seed liquid was incubated into PDB with or without FG for 2 days at 30°C under shaking conditions. The mycelium of P5 with and without FG were collected after 12 h incubation. The mycelium was then centrifuged at 12,000 rpm, 4°C for 10 min in an RNase free tube, and washed three times with 20 mM potassium phosphate buffer (pH 7.2). Transcriptome experiments were undertaken in three parallel samples. The mycelium pellets were stored at −80°C prior to analysis (Tan et al., 2020).

### RNA Isolation, cDNA Library Preparation, and Transcriptome Sequencing

Total RNA was extracted with cDNA library preparation in accordance with the method described by Zhang et al. (2020). The transcriptome was sequenced using an Illumina NovaSeq 6000 (150bp^∗^2, Shanghai BIOZERON Co., Ltd.). To identify differential expression genes (DEGs) between two different samples, the expression level of each transcript was calculated according to the reads per kilobase of exon per million mapped reads (RPKM). The fold change of DEGs was shown as the log_2_ Fold Change (log_2_ FC) of gene abundance through comparison of the FG stimulated sample and the control group; and the primary screening criterion was log_2_ FC ≥ 1.

### LC-HRMS Analysis

The metabolites formed during biodegradation of RB4 were extracted from the cell free supernatant by using resin XAD-16 absorption then eluted by methanol and concentrated using a rotary evaporator for further analysis. The RB4 dye and its degraded products were evaluated by LC-HRMS chromatography. The degraded metabolite dyes were analyzed using LC-MS (Agilent 6546 LC/Q-TOF).

HPLC analysis was carried out with a ZORBAX C18 column (50 mm × 2.1 mm Agilent Technologies Inc., CA, United States). The binary mobile phase used in the experiment was composed of (A) water (0.1% FA) and (B) methanol gradient with the following elution program: 0–0.5 min, 90% A; 0.5–10 min, 90-0% A; 10–13 min, 0% A; 13–16 min, 0-90% A; 13–20 min, 90% A. The MS interphase is equipped with electron spray ionization (ESI) in both positive and negative ionization modes. The samples were eluted at a flow rate of 0.3 mL min^–1^ and monitored at 280 nm. For MS analysis, N2 was used as the nebulizing gas (35 psig), heated sheath gas (350°C), and drying gas (10 L min^–1^). The method of HPLC and MS analysis was based on previous research (Navada et al., 2018; Alam et al., 2020).

### Acute Toxicity Assessment

The acute toxicity of the target dye before and after decolorization was assessed by *Vibrio fischeri* powder purchased from J and Q Environmental Technologies Co., Ltd. (Beijing, China). The assay method followed Qiu et al. (2016). For the acute toxicity test, *Vibrio fischeri* powder was recovered using 3% NaCl solution at 20°C for 15 min. After 15 min exposure to the samples in a 96-well microplate, the relative luminescence intensity was determined by PerkinElmer VICTOR X3. Samples were tested in triplicate with one blank control. Toxicity was evaluated by the inhibition ratio, which was calculated by the following equation: IR = (1-LU/LU_0_) ^∗^100%, where LU is the relative light unit (RLU) of *Vibrio fischeri* exposed to the experimental group, and LU_0_ is the RLU to the blank control. Samples were tested in triplicate with one blank control.

### Statistical Analysis

Measured values were expressed as the mean ± standard deviation. Statistical determination via t-tests and analysis of variance (ANOVA) was implemented in GraphPad Prism software. There was a statistically significant difference when *P* < 0.05.

## Results and Discussion

### Identification and Decolorization Characteristics of P5

**FIGURE 1:**
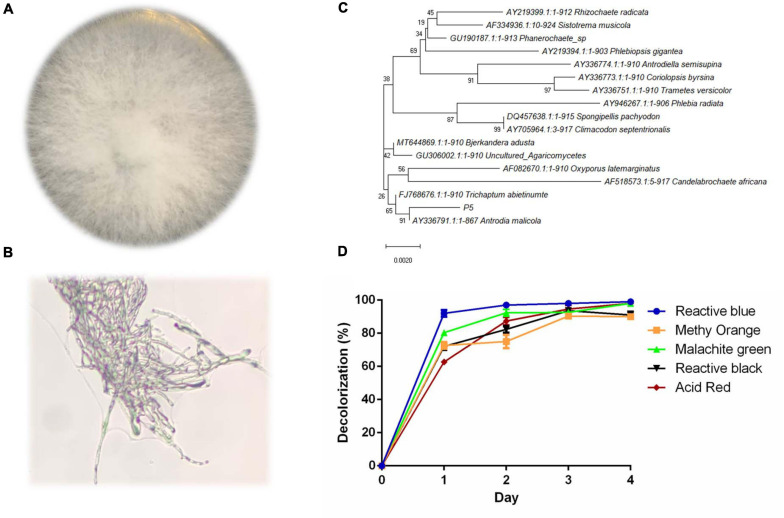
Identification and decolorization characteristics of P5 **(A)** Colony morphology of P5. **(B)** Microscopic morphology of P5 with a 40X objective lens. **(C)** Neighbor joining phylogenetic tree analysis of P5. **(D)** Color removal efficiency of five different dyes by P5.

The colony of strain P5 was round and the mycelium was white and strong; there was no obvious pigment produced on the PDA plate (Figures 1A,B). The 18s rDNA partial sequence of strain P5 with a length of 950 bp was amplified by PCR and sequenced. The sequenced result was deposited in the GeneBank database with accession number MW227322. According to the phylogenetic tree in Figure 1C, P5 exhibits high homology to the white rot fungus *Antrodia*. As noted above, five different kinds of dyes including Reactive Blue4, Methyl Orange, Malachite Green, Reactive Black 19, and Acid Red were selected to estimate the decolorization ability of P5. The color removal percentages of the five dyes were all above 80% under 4 days’ biodegradation (Figure 1D), which indicated that P5 has strong decolorization potency on a wide range of industrial dyes. Among the five dyes, RB4 was the most efficiently decolorized by P5 and was therefore selected as the target dye for further investigation.

### Effect of Varying Physicochemical Parameters on RB4 Decolorization by P5

**FIGURE 2:**
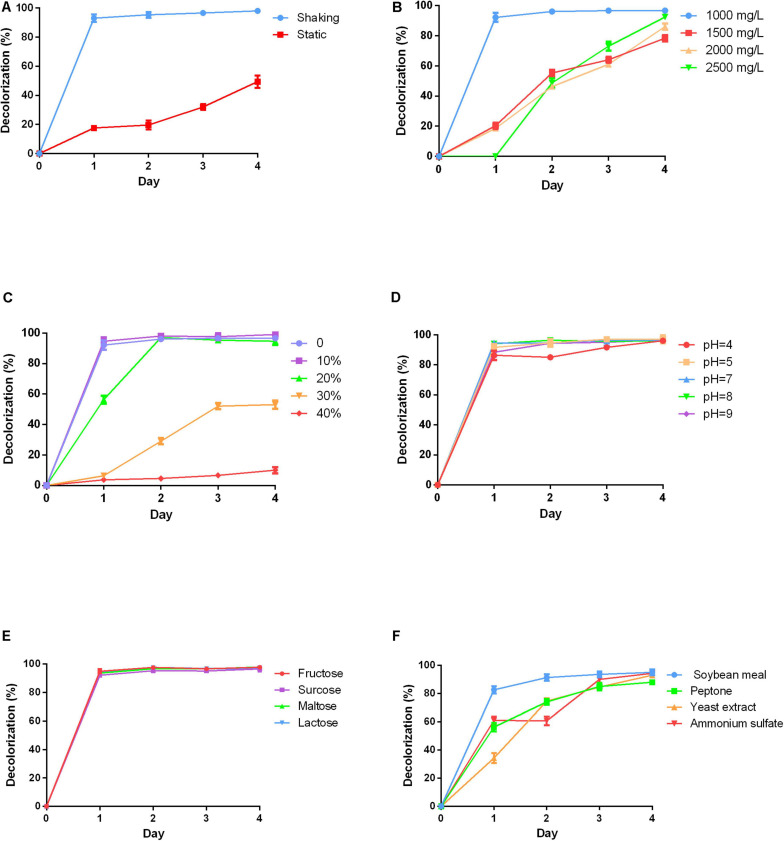
Effects of varying physicochemical parameters on RB4 decolorization by P5 **(A)** RB4 decolorization under shaking and static conditions. **(B)** RB4 decolorization under different initial dye concentrations. **(C)** RB4 decolorization under different salt concentrations. **(D)** RB4 decolorization under different pH environments. **(E)** RB4 decolorization by adding different carbon sources. **(F)** RB4 decolorization by adding different nitrogen sources.

Anthraquinone dyes are relatively recalcitrant in the environment due to their bulky aromatic structure. The culture conditions for RB4 decolorization efficiency by P5 were firstly investigated. The results suggested that color removal efficiency was significantly increased under shaking conditions compared with static conditions as shown in Figure 2A. This can be attributed to the aerophilic nature of white rot fungus. In shaking conditions, RB4 was efficiently removed in 24 h, which emphasizes the roles played by oxygen in the removal of anthraquinone dyes.

High concentrations of dyes are toxic to fungus. A range of initial dye concentrations was investigated. Nearly 95% of RB4 was decolored at a concentration of 1,000 mg/L in 24 h by P5. As the initial concentration increased, the time required to reach the maximum decolorization percentage also increased. When the concentration of RB4 increased to 2,000 mg/L, the decolorization rate declined to 20% in 24 h (Figure 2B). However, P5 has a strong tolerance capacity toward RB4 with the highest effective biodegradation concentration reaching 2,500 mg/L.

Industrial dye wastewater usually contains a certain percentage of salts, which may adversely affect the efficiency of microbial bioremediation of dyes. Therefore, different concentrations of NaCl were investigated. P5 could still maintain efficient biodegradation performance under a 10% NaCl concentration as displayed in Figure 2C. When the salt concentration was increased to 20%, the maximum removal efficiency was accordingly delayed to 48 h. However, higher salinity environments (30–40%) significantly inhibited the decolorization process, and as such may be hazardous for P5 growth. Different pH conditions were also explored. P5 was well adapted to a wide range of pH conditions (4–9) and the efficient biodegradation performance was unaffected by acid and alkaline environments as shown in Figure 2D.

Carbon is essential for fungal growth, as it also supplies oxidants which mediate the decolorization of dyes. Four different carbon sources including fructose, sucrose, maltose, and lactose were added into the PDB to explore their effects on dye removal efficiency. The addition of the extra carbon sources did not change the dye removal rate. All the carbon sources were associated with similar decolorized efficiency as shown in Figure 2E, which indicates good utilization of different carbon sources by P5. The effects of different nitrogen sources including soybean meal, peptone, yeast extract, and ammonium sulfate on dye removal efficiency were also investigated (Figure 2F). The results showed that supplementing extra nitrogen sources significantly decreased dye removal efficiency. This suggests that an inappropriate C/N ratio may impede the growth and metabolic pathways of P5.

Overall, P5 was able to efficiently biodegrade RB4 under basic culture conditions. Oxygen is essential for fungus growth and provides the energy for fungal metabolic activities. Our results indicated that simply under shaking conditions with a good supply of oxygen, P5 could efficiently biodegrade the dye. Compared with other microorganisms that can biodegrade anthraquinone dye, P5 showed a stronger tolerance to RB4: the effective degradable concentration was up to 2,500 mg/L. However, other studies exploring microbial treatment of RB4 concentration have tended to use dye concentrations lower than 500 mg/L (Li et al., 2019). The concentration of dye discharged from textile factories differs significantly, depending on the process, equipment, and type of dye produced (Brik et al., 2006). P5 possessed good adaptability to a wide range of dye concentrations. However, actual effluents from the textile industry usually contain different kinds of dyes. P5 was observed to efficiently degrade five different dyes, which indicates that it could be leveraged in a broad array of practical contexts.

Industrial dye wastewater contains 5–10% salt and the pH environment fluctuates between 4 and 10 (Yaseen and Scholz, 2019). Our results indicated that the dye biodegradation process of P5 was unimpeded under the mimetic industrial environment of 10% NaCl concentration and a wide pH range. However, actual dye wastewater also contains various metals and organic solvents, which should be investigated in follow-up experiments for possible industrial applications in the future. Importantly, carbon and nitrogen sources are often applied to provide nutrition for promoting microbial growth and enzyme production. However, economical nutrients are often chosen for microbial biodegradation in practical treatment systems, which can result in poor decolorization performance. P5 showed effective dye biodegradation with simple medium components, which could reduce production costs. On the other hand, oversupply of carbon and nitrogen sources can also negatively affect the efficacy of dye removal (Zheng et al., 2020); the balance of the C/N ratio could play an important role in the decolorization process.

### The Effect of HER on RB4 Biodegradation by P5

**FIGURE 3:**
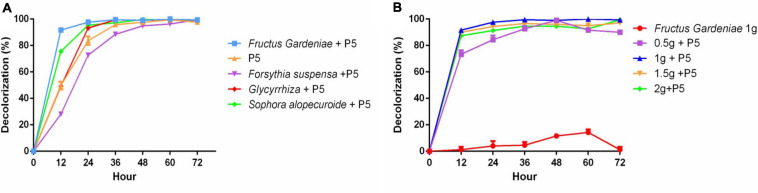
The effect of HER on RB4 biodegradation by P5 **(A)** RB4 decolorization by adding different HERs **(B)** RB4 decolorization under different concentrations of FG.

Given the substantial and increasing demand for Chinese herbal medicine, HER as industrial waste puts substantial pressure on the environment. Thus there is an urgent need to recycle HER. In our study, four different HERs including *Fructus Gardeniae* (FG), *Glycyrrhiza uralensis* (GU), *Forsythia suspensa* (FS), and *Sophora alopecuroides* (SA) were selected as medium elements to explore their effect on RB4 biodegradation by P5. These four HERs were added into PDB and cultured with P5 for 48 h before adding RB4. Interestingly, the addition of FG significantly improved the dye removal performance of RB4 in 12 h (Figure 3A). The dye removal rate increased from 47% to 92%, compared with P5 without FG supplementation. The effect of additive amounts of FG on dye removal efficiency was also estimated (Figure 3B) and the results showed that a supplementary amount of FG between 10 g/L and 20 g/L was associated with effective dye removal efficiency. It has hitherto been reported that the extraction residue of *Salvia miltiorrhiza Bge* can directly absorb methylene blue from waste water (Zhao and Zhou, 2016). Our absorption assay showed that 10% RB4 was absorbed after 48 h incubation with only the addition of FG (Figure 3B); this demonstrates that the enhanced decolorized performance of RB4 following FG treatment could be attributed to a biochemical reaction rather than physical absorption.

### The Possible Mechanism of FG Enhancing Dye Removal Performance

It is well known that different culture media have a direct impact on the metabolic activity of microorganisms and this can be analyzed through untargeted-metabolomics, quantitative proteomics, or transcriptome sequencing (Lv et al., 2019; Zhang Q. Y. et al., 2019; Zheng et al., 2020). To explore the possible mechanism of dye enhanced decolorization by addition of FG, differentially expressed genes were analyzed by comparative transcriptome analysis in this study. Three parallel samples of the control group without FG and the FG addition group were collected when RB4 was biodegraded for 12 h. The raw transcriptome sequence data were uploaded into NCBI database with BioProject accession number PRJNA675897. In total, 8671 genes were differentially expressed between the two groups. There were 5,290 upregulated genes in the FG addition group (Supplementary Figure 1).

After the detailed examination of differential genes and pathway expression, the genes involved in the possible mechanism of enhanced dye removal performance by FG were generally divided into three groups. The first group was extracellular non-specific peroxidase, including LiP, MnP, Lac, and DyP, which have been considered as the main participants in the biodegradation process of anthraquinone synthetic dyes (Hsu et al., 2012; Uchida et al., 2015; Navada et al., 2018; Sekuljica et al., 2020). Previous research has tended to focus on a single enzyme involved in the dye biodegradation process. However, our results showed that gene expression of multiple peroxidases increased significantly under the addition of FG. In total, 10 LiPs, 8 MnPs, 2 Lacs, and 1 DyP increased remarkably when supplemented with FG; the maximum increase of LiP gene (TRINITY_DN12305) reached 10.22-fold (Table 1). Our results indicated that P5 was rich in various peroxidases, which is consistent with its strong dye removal ability. Moreover, LiPs and MnPs were considered as the major enzymes responsible for the dye decolorization ability of P5 that was significantly stimulated by the addition of FG.

**TABLE 1:** Differentially expressed genes related to dye biodegradation peroxidases under stimulation with FG.

Description	Gene ID	Enzyme	Log FC	*P* value
Extracellular peroxidase	TRINITY_DN10596	LiP	2.94	1.50E-12
	TRINITY_DN10638	LiP	2.02	0.0020
	TRINITY_DN10959	LiP	3.65	1.92E-06
	TRINITY_DN12305	LiP	10.22	1.15E-15
	TRINITY_DN14131	LiP	5.12	6.27E-20
	TRINITY_DN5455	LiP	3.03	3.90E-06
	TRINITY_DN6524	LiP	3.15	0.0002
	TRINITY_DN7607	LiP	2.20	0.0100
	TRINITY_DN8761	LiP	1.55	0.0080
	TRINITY_DN9117	LiP	1.77	4.60E-05
Extracellular peroxidase	TRINITY_DN11138	MnP	1.54	0.00196
	TRINITY_DN11292	MnP	2.55	7.21E-09
	TRINITY_DN13293	MnP	5.10	2.84E-16
	TRINITY_DN6386	MnP	1.79	0.0001
	TRINITY_DN7864	MnP	3.57	0.0001
	TRINITY_DN8914	MnP	3.33	4.11E-16
	TRINITY_DN9482	MnP	3.48	6.32E-08
Extracellular peroxidase	TRINITY_DN13976	DyP	4.25	9.30E-06
Extracellular peroxidase	TRINITY_DN15241	Lac	3.16	4.78E-17
	TRINITY_DN5241	Lac	1.72	0.0236

The second group of genes was related to the oxidative reaction and radical generation (Sun et al., 2016). Essentially, peroxidases require reactive oxygen and other radicals for dye decolorization and aromatic degradation. These genes mainly included alcohol dehydrogenase, glucose oxidase, and cellobiose dehydrogenase (Table 2). The oxidase directly reacts on the alcohol and glucose to generate reactive oxygen such as H_2_O_2_, which provides the redox potential facilitating the peroxidase to catalyze the dye biodegradation. Cellobiose dehydrogenase can generate hydroxyl radicals, which could also synergistically assist with manganese peroxidase activity (Hilden et al., 2000). It was noticeable that under FG treatment, these oxidases were induced to a higher expression level to assist efficient dye degradation.

**TABLE 2:** Differentially expressed genes related to oxidative reactions and radical generation under stimulation with FG.

Description	Gene ID	Enzyme	Log FC	*P* value
Oxidative reactions and radical generation	TRINITY_DN15192	Aryl-alcohol oxidase	12.99	2.63E-26
	TRINITY_DN12771	Aryl-alcohol dehydrogenase dehydrogenase	1.75	0.0071
	TRINITY_DN1940	Aryl-alcohol dehydrogenase	2.65	0.018
	TRINITY_DN2584	Aryl-alcohol dehydrogenase	2.09	0.01
	TRINITY_DN6101	Aryl-alcohol dehydrogenase	1.78	0.02
	TRINITY_DN6760	Aryl-alcohol dehydrogenase	2.92	0.006
	TRINITY_DN14364	Glucose oxidase oxidase	3.34	4.04E-09
	TRINITY_DN13964	Cellobiose dehydrogenase	2.96	1.01E-05

In the last group, the genes involved in co-factors were enriched in the FG treatment samples (Table 3). The iron transporter and iron permease were significantly upregulated under supplementation with FG, consistent with the high expression of LiP; this suggests that LiP could play an important role in the biodegradation of RB4 by P5. Moreover, the Di-copper center-containing protein related to Lac activity was also substantially upregulated. One gene, DN12243, encoding salicylate hydroxylase (E.1.14.13.1) involved in polycyclic aromatic hydrocarbon degradation (Supplementary Figure 2) was upregulated 2.55-fold, and thus might also play an important role in the improvement of dye biodegradation performance.

**TABLE 3:** Differentially expressed genes related to iron transportation and other biological processes under stimulation with FG.

Description	Gene ID	Enzyme	Log FC	*P* value
Genes involved in iron transportation	TRINITY_DN11656	Iron transporter	1.87	0.016
	TRINITY_DN14644	Iron permease	1.89	5.07E-05
	TRINITY_DN1135	Di-copper center-containing protein	4.72	7.27E-29
	TRINITY_DN15704	Di-copper center-containing protein	3.09	3.34E-08
Other processes	TRINITY_DN12243	Salicylate hydroxylase	2.55	1.19E-06

**FIGURE 4:**
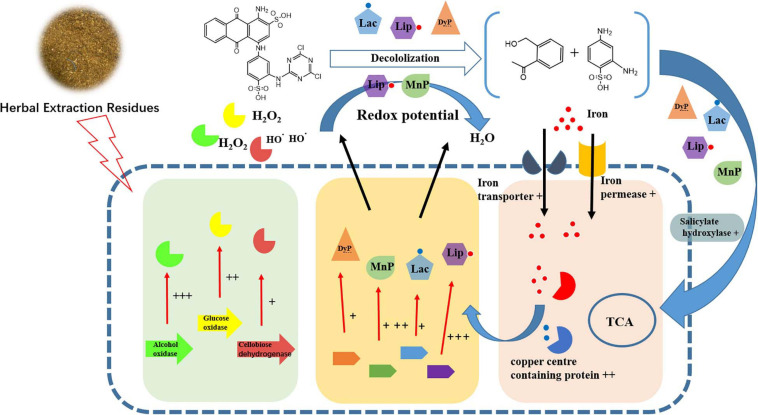
Schematic diagram of changes in transcript abundance of genes involved in RB4 biodegradation by P5 in response to the addition of FG. + + + indicates gene expression of LogFC > 5, + + indicates gene expression of LogFC > 3, and + indicates genes expression of LogFC > 1.

It was noted that the main components of HER were cellulose and lignin. Moreover, the solid structure of HER is destroyed after the extraction and autoclave process under high temperature and pressure conditions (Wang et al., 2018). It is suggested that under HER stimulation, P5 was induced to express a complex network (Figure 4) including multiple peroxidases, redox potential generating enzymes, and other accessory enzymes to achieve efficient RB4 decolorization.

### The Proposed RB4 Biodegradation Pathway by P5

**FIGURE 5:**
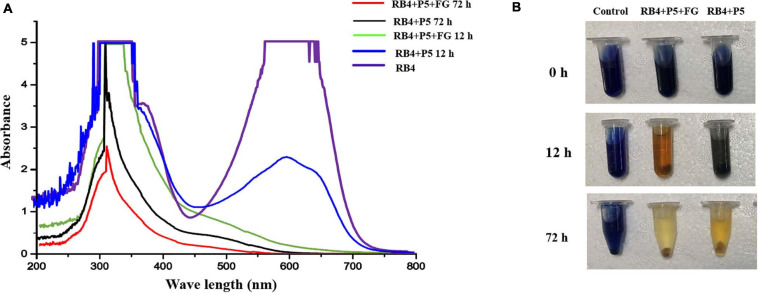
UV–Vis absorbance spectra and images of RB4 biodegradation by P5. **(A)** UV scanning results of the biodegradation process with and without FG. **(B)** Images of RB4 biodegradation solution with and without FG.

**FIGURE 6:**
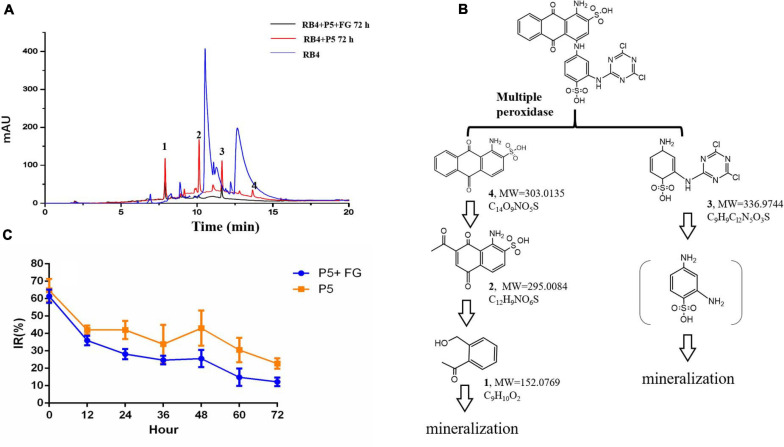
LC-MS analysis and toxicological effects of biodegraded metabolites of RB4 **(A)** HPLC analysis of biodegraded metabolites of RB4 with and without FG. **(B)** Proposed pathway of RB4 biodegradation by P5. **(C)** Acute toxicological effect analysis of RB4 biodegraded metabolites.

Based on the decolorization results, RB4 was removed by the biodegradation process of P5. The UV scanning results showed that the intensity at 595 nm (λ_max_ of RB4) significantly decreased, which indicated disruption of the anthraquinone dye structure. When P5 was supplemented with FG, the maximum absorbance of RB4 decreased substantially at 12 h, and there was a dramatic color change (Figures 5A,B). Most of the absorbance at 595 nm disappeared when biodegraded for 72 h, with or without FG. However, the absorbance around 310 nm was significantly reduced only with FG supplementation. Furthermore, LC-HRMS was applied to analyze possible metabolites and concentration changes (Figure 6A).

Our results revealed that the parent RB4 was completely disrupted into three major intermediates after biodegradation for 72 h and its m/z values are as follows: metabolite 1 (m/z = 152.0769, C_8_H_9_O_2_, Supplementary Figure 3), metabolite 2 (m/z = 295.0085, C_12_H_9_NO_6_S, Supplementary Figure 4), metabolite 3 (m/z = 336.9744, C_9_H_9_Cl_2_N_5_O_3_S, Supplementary Figure 5), and a minor peak of metabolite 4 (m/z = 303.0135, C_14_O_9_NO_5_S, Supplementary Figure 6) was identified as the anthrone part of RB4. The proposed RB4 biodegradation pathway by P5 as described in Figure 6B is consistent with Chaudhari et al. (2017).

Generally, anthraquinone dye biodegradation results from a biochemical reaction that breaks down the conjugated dye bonds (Li et al., 2019). The first degradation step that occurred was hydrolysis of C–N in the dye structure. It was noted that white rot fungus P5 could secrete multiple oxidoreductase enzymes which efficiently dissociate the side chain from the anthraquinone ring. The residual polycyclic aromatic structures are further decomposed into single rings or smaller compounds by oxidation and hydrolysis. Phthalic acid is an oxidation product of compounds with an anthraquinone structure (Panizza and Cerisola, 2009). The aromatic hydrocarbons will finally breakdown into carbon dioxide and water and the side chain of anthraquinone dyes also goes through biodegradation. The intermediates of the side chain can form the polymer compound 2,2′-disulfonyl azo benzene under the action of DyP (Sugano et al., 2009). Hydrolysis also plays an important role in side chain degradation which may finally generate cyanuric acid, a non-toxic compound (Alam et al., 2020).

Our results showed that RB4 was mainly biodegraded into three intermediates by P5; metabolites 3 and 4 were the side chain and anthraquinone part of RB4, respectively. It was observed that the relative abundance of metabolite 4 was much lower than other metabolites, which indicated that the anthraquinone part of RB4 could be efficiently biodegraded into smaller aromatic compounds by the peroxidase from P5. Moreover, the concentrations of RB4 biodegraded metabolites 1, 2, and 3 were significantly reduced in the FG addition group compared with the control group, which indicated that FG could promote higher expression of peroxidase for the synergistic decomposition of refractory polycyclic aromatic compounds.

### Acute Toxicological Assay of RB4 Biodegraded Metabolites

There is no doubt that the direct release of dyes into water bodies influences human health. Additionally, the breakdown of dyes may also generate toxic intermediates. Therefore, the acute toxicological effects of RB4 and its biodegradation intermediates was evaluated using *V. fischeri*. Our results showed that 1,000 mg/L RB4 was highly toxic to *V. fischeri*. After the biodegradation treatment by P5, the toxicological effects of RB4 decreased to lower levels. The inhibition rate was significantly decreased to only 10% following supplementation with FG when RB4 was biodegraded for 72 h (Figure 6C). The abatement of RB4 toxicity reflected the synergistic effect of FG supplementation, which accelerated the decomposition of the toxic molecules. It has been reported that anthraquinone dye biodegradation intermediates are more toxic than the original dye compound (Champagne and Ramsay, 2010). However, our results showed that along with the RB4 decolorization process, the toxic effect of RB4 was also effectively reduced. Therefore, treatment of RB4 with P5 under the supplementation of FG may provide an effective and promising strategy for the management of industrial dye wastewater.

## Conclusion

One new white rot fungus named P5 was recently isolated and characterized. It can efficiently biodegrade 95% 1,000 mg/L of RB4 in 24 h under the following conditions: Potato Dextrose Broth, shaking at 160 rpm, 30°C, pH 4–9, and 10% salt concentration. Moreover, herbal extraction residue was applied in the RB4 biodegradation context: the addition of 1% (m/v) *Fructus Gardeniae* residue significantly promoted biodegradation performance with 92% decolorization in 12 h. The comparative transcriptome sequencing results revealed that the expression of multiple peroxidase genes was simultaneously increased. Lignin Peroxidase increased 10.22-fold under the stimulation of FG. P5 was induced to operate a synergetic network including multiple peroxidases, redox potential generating enzymes, and other accessory enzymes to achieve efficient RB4 degradation under stimulation with FG. Fungal treatment with herbal extraction residues can significantly accelerate the biodegradation of RB4 intermediates, as well as alleviate toxic effects. This therefore provides important insights into a novel treatment for industrial dye wastewater.

## Data Availability Statement

The datasets presented in this study can be found in online repositories. The names of the repository/repositories and accession number(s) can be found in the article/Supplementary Material.

## Author Contributions

TY conceived the study. SZ, YC, and RZ carried out the sample collection, data analysis, and validation of the results. SZ and XR provided the herbal extraction residues. TY and LC wrote the paper. FZ helped to revise the paper. All authors read and approved the final manuscript.

## Conflict of Interest

The authors declare that the research was conducted in the absence of any commercial or financial relationships that could be construed as a potential conflict of interest.
