# Supplementary material for: Enhanced Reactive Blue 4 Biodegradation Performance of Newly Isolated white rot fungus Antrodia P5 by the Synergistic Effect of Herbal Extraction Residue
Source: Front Microbiol. 2021 Mar 30;12:644679. doi: 10.3389/fmicb.2021.644679 (PMC8044803; doi:10.3389/fmicb.2021.644679)
Supplement: Supplementary file 1 [file Data_Sheet_1.DOCX]

Supplementary Material

**Enhanced Reactive Blue 4 Biodegradation Performance of Newly Isolated *white rot fungus P5* by the synergistic effect of Herbal Extraction Residue**

Tianjie Yuan^a*^, Shuyi Zhang^a^, Yifei Chen^a^, Ran Zhang^a^, Letian Chen^a^, Xiaoshu Ruan^a^, Sen Zhang^a,b^ , Fang Zhang^a,b^

^a^ School of Pharmacy, Nanjing University of Chinese medicine, Nanjing, 210023, China

^b^ Jiangsu collaborative innovation center of Chinese medical resources industrialization, Nanjing University of Chinese medicine, Nanjing, 210023, China

*Correspodning author: Tianjie Yuan

*E-mail address*: YTJ@njucm.edu.cn


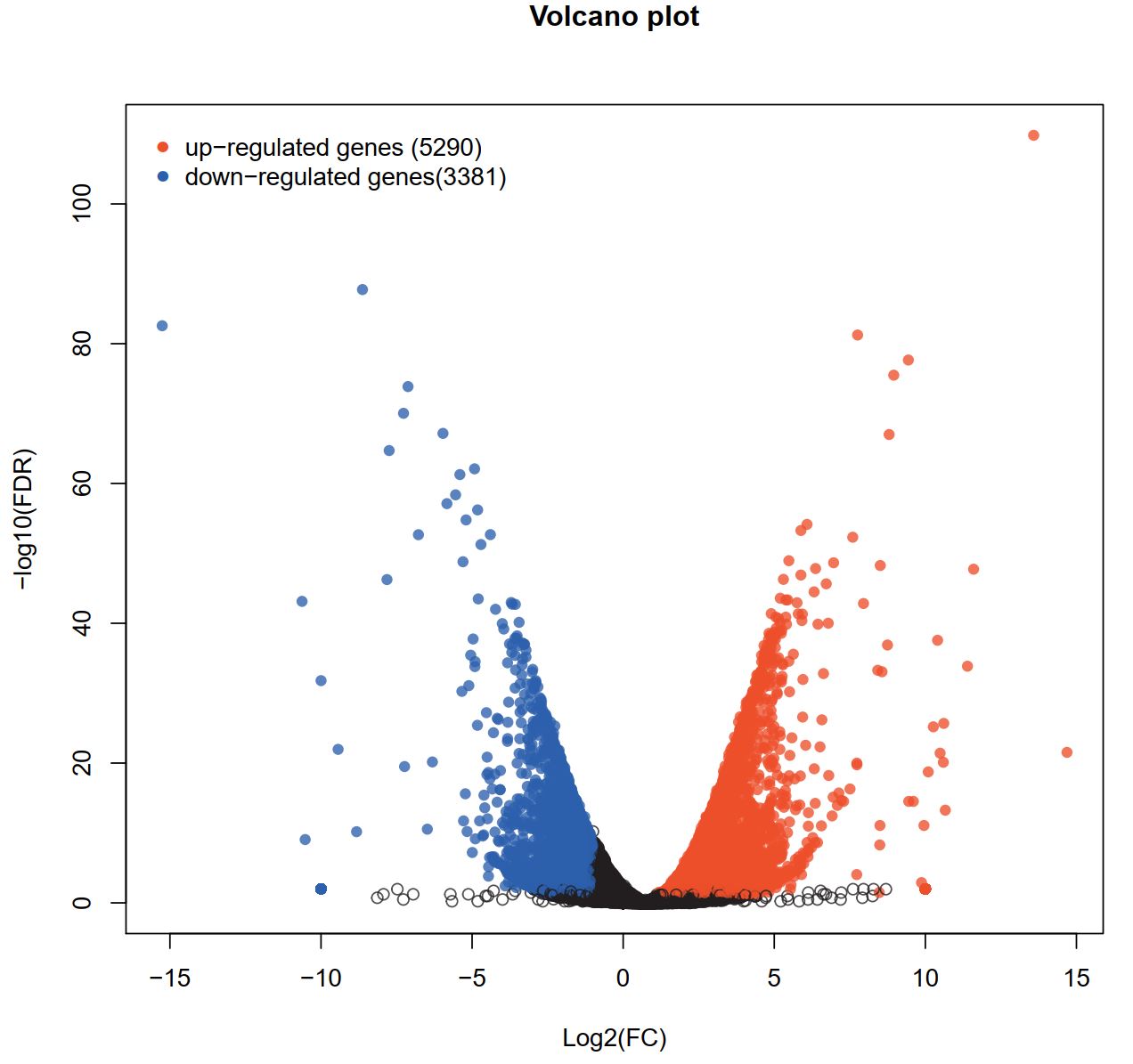


**FIGURE S1** Volcano map of differential expression genes between experimental group (P5 with HER) and control (P5). Significantly differential expression genes are shown as a red (up) or blue(down) dot. No significant difference between the expressions of genes is shown as a black dot.


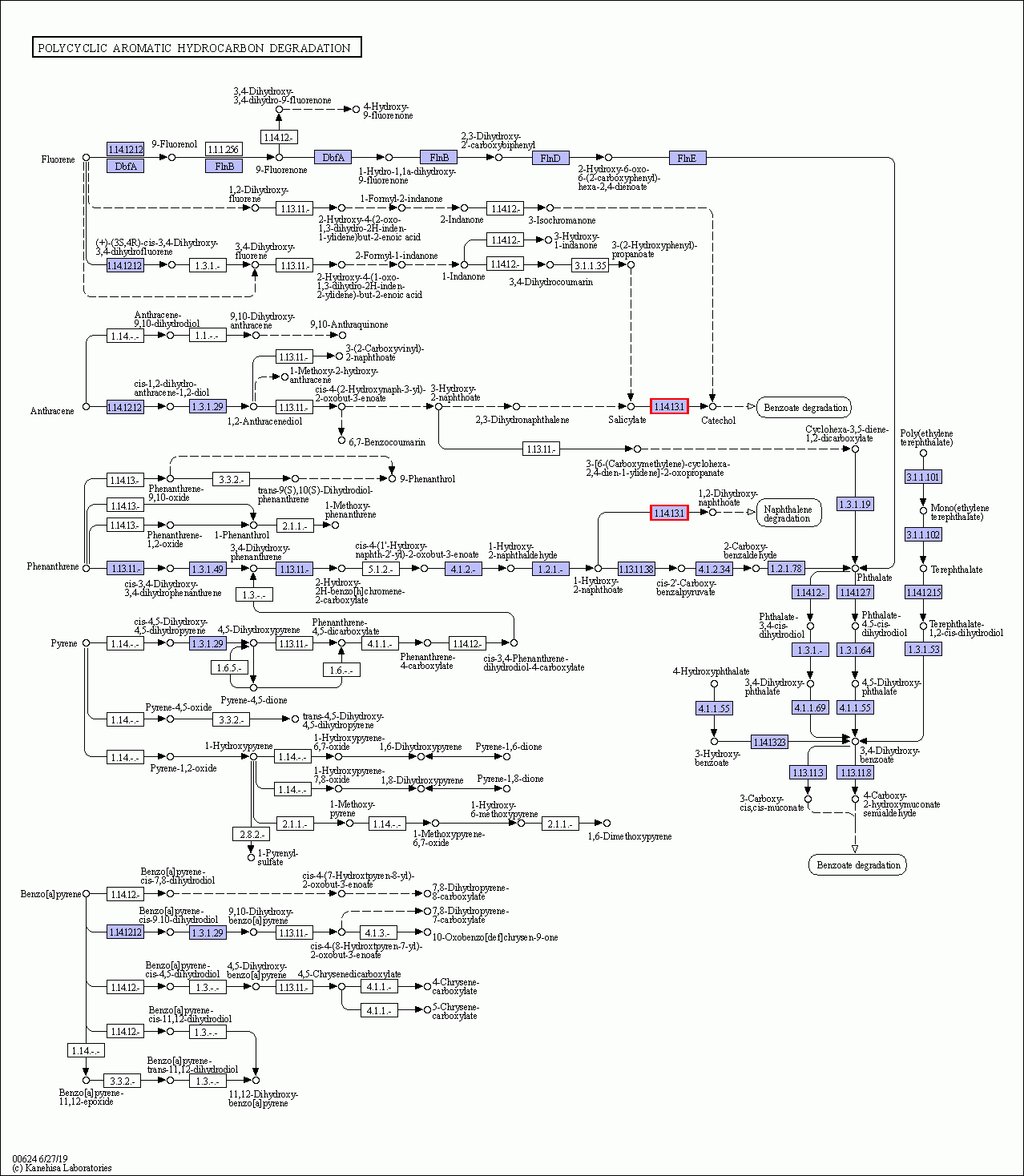


**FIGURE S2** Comparative transcriptome analysis for the related genes of polycyclic aromatic hydrocarbon degradation pathway the with and without HER addtion. The red frame means significantly up-regulated genes The pathway maps were adapted from KEGG pathway database


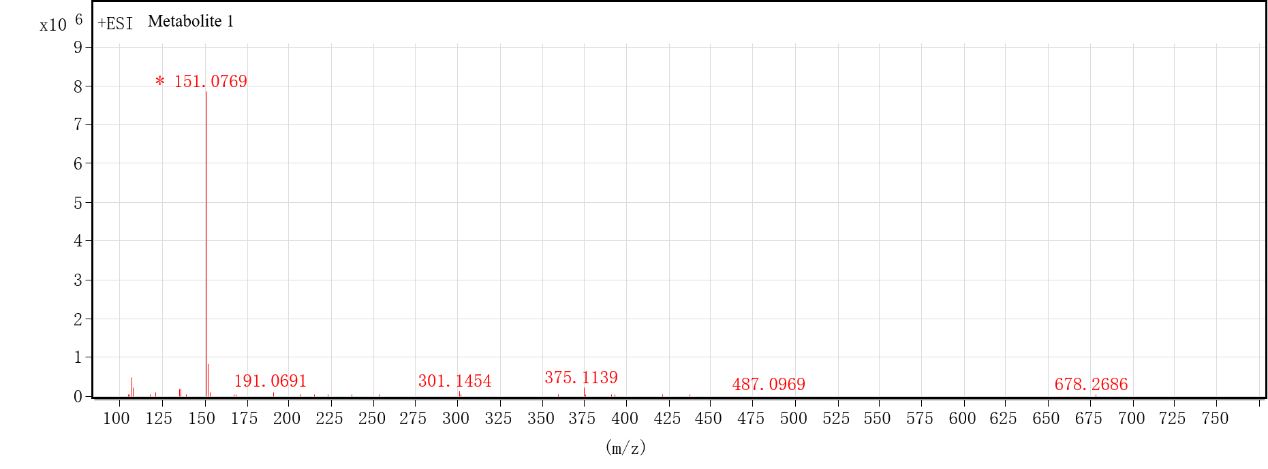


**FIGURE S3** HRMS spectra of RB4 biodegradation metabolite 1


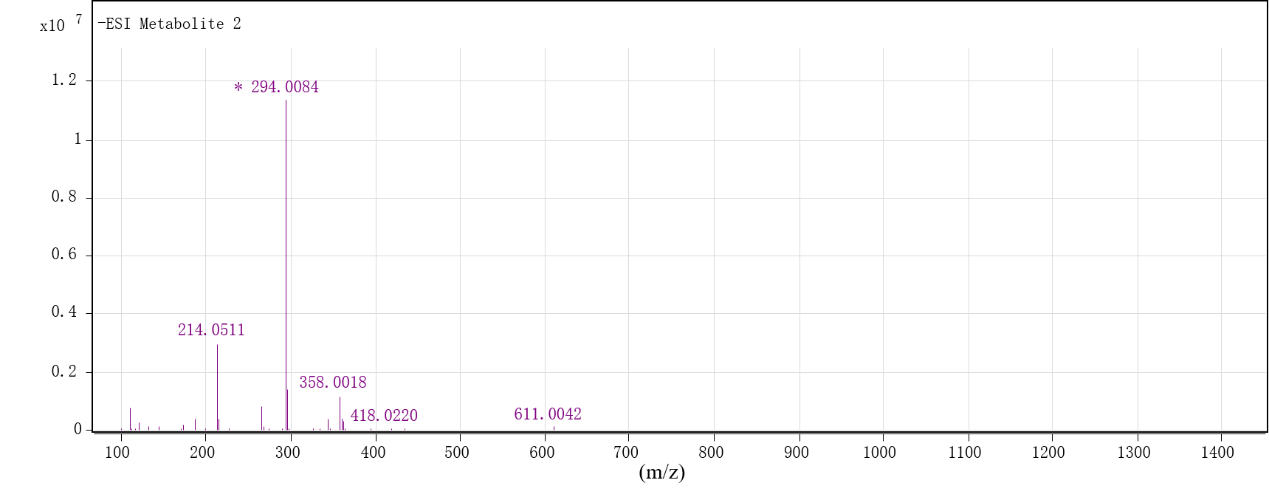


**FIGURE S4** HRMS spectra of RB4 biodegradation metabolite 2


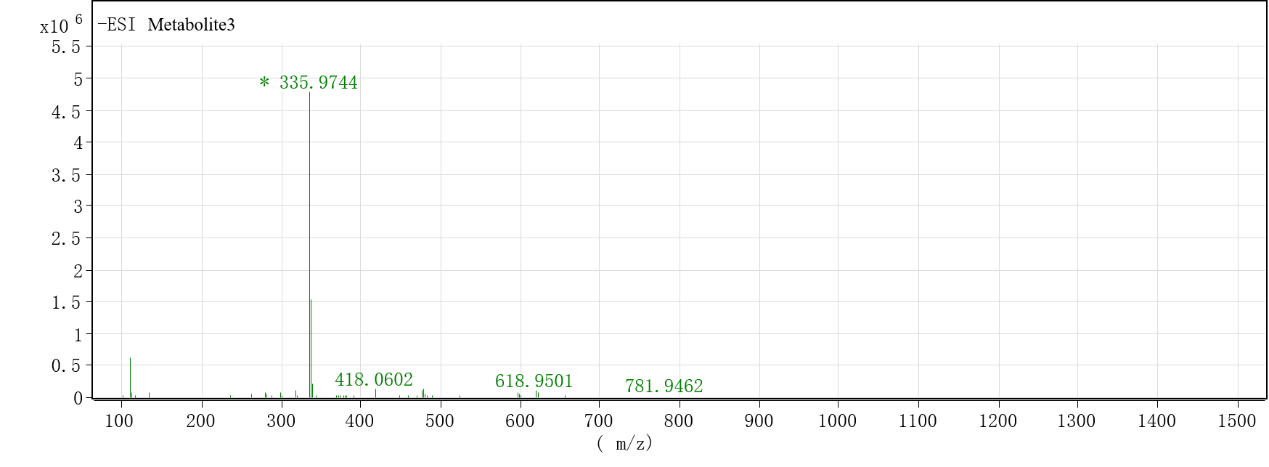


**FIGURE S5** HRMS spectra of RB4 biodegradation metabolite 3


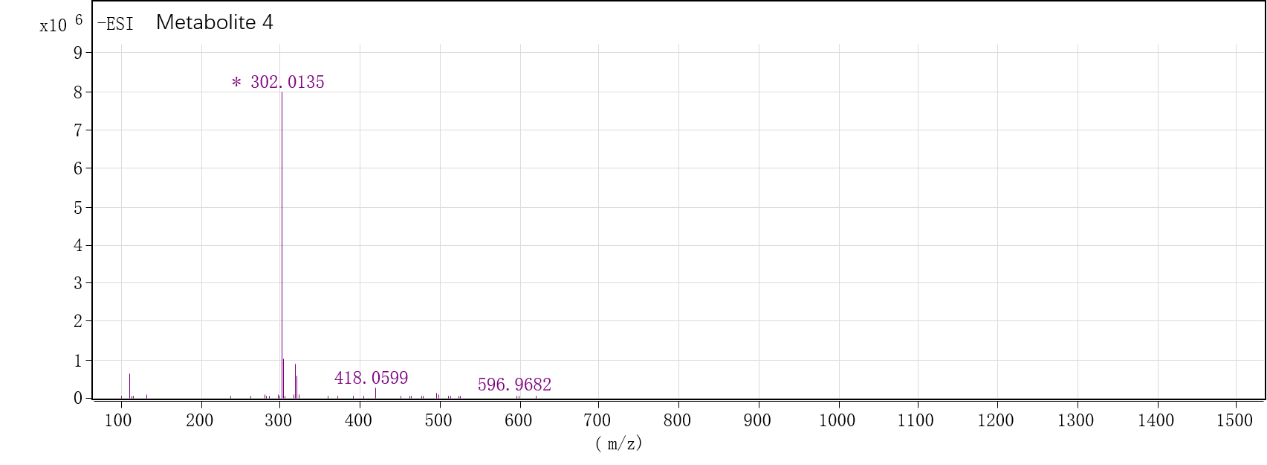


**FIGURE S6** HRMS spectra of RB4 biodegradation metabolite 4

# Supplementary Tables

Supplementary Table 1. Primer sequence and annealing temperature for Real-time PCR

| Gene | Forward sequence (5′–3′) | Reverse sequence (5′–3′) | Annealing temperature |
| --- | --- | --- | --- |
| GAPDH | TCCTGCACCACCAACTGCTTAG | AGTGGCAGTGATGGCATGGACT | 56.3℃ |
| MDR1 | TCTTGAAGGGCCTGAACCTG | AGTCATAGGCATTGGCTTCC | 56.3℃ |
| MRP-2 | TGAGCAAGTTTGAAACGCACAT | AGCTCTTCTCCTGCCGTCTCT | 58.3℃ |
| MRP-4 | GCTCAGGTTGCCTATGTGCT | CGGTTACATTTCCTCCTCCA | 52℃ |
| BCRP | TGCAACATGTACTGGCGAAGA | TCTTCCACAAGCCCCAGG | 56.3℃ |
